# Ion-Selective Electrodes for Ammonium and Nitrate Determination: Recent Advances, Trends and Perspectives

**DOI:** 10.3390/ijms27104432

**Published:** 2026-05-15

**Authors:** Klaudia Morawska, Cecylia Wardak

**Affiliations:** Department of Analytical Chemistry, Faculty of Chemistry, Institute of Chemical Sciences, University of Maria Curie-Skłodowska, Maria Curie-Sklodowska Sq. 3, 20-031 Lublin, Poland

**Keywords:** mineral nitrogen, nitrates, ammonium, ion-selective electrodes, potentiometry, review

## Abstract

In response to increasing environmental protection requirements and the rapid advancement of analytical technologies, particular attention is being paid to the development of environmentally friendly, cost-effective, and sensitive measurement tools. One of the key challenges in modern analytics is the effective monitoring of inorganic nitrogen in the natural environment. In this review, we present a variety of ammonium and nitrate ion-selective electrodes. The review includes the years 2020–2026. It was divided into sections based on sensitivity to a given ion, as well as into subsections based on application: monitoring of soil and aquatic environments, the use of sensors for the determination of specific ions in a wide range of samples, and electrode designs that were used only in laboratory studies. A total of 64 nitrate electrodes and 22 ammonium electrodes were analyzed. Comparisons were made based on electrode type, type of internal contact, materials used to enhance ion-to-electron conductivity, and type of ionophore. Additionally, analytical parameters were compiled, including sensitivity, detection limit, linearity range, potential stability, operating pH range, and actual application scenarios. This review allows for an assessment of current trends and may serve as a basis for the design of new potentiometric sensors.

## 1. Introduction

**Figure 1:**
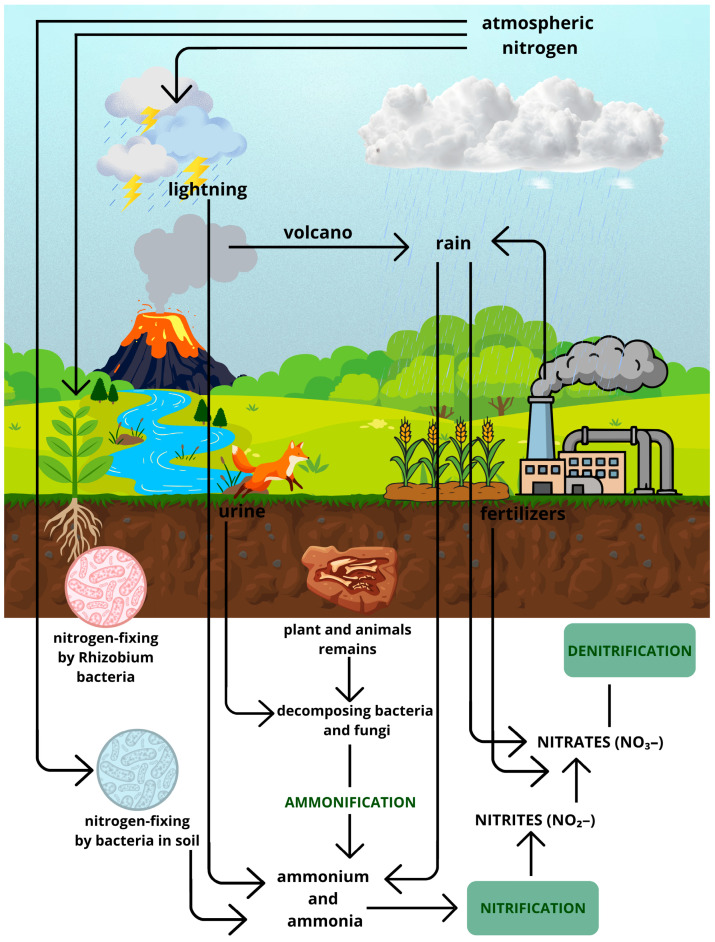
Scheme of biogeochemical nitrogen cycle.

Nitrogen is one of the most important elements on our planet. Nitrogen can originate from natural sources—such as mineralization of organic matter, nitrification, denitrification, volcanic ash, and lightning—as well as from anthropogenic sources, such as intensive agriculture and increased fertilizer use, wastewater, and industrial expansion [1]. It serves many functions, but above all, it plays a key role in the biogeochemical cycle, as it is essential for the proper functioning of ecosystems [2,3]. The nitrogen cycle consists of several stages: (1) nitrogen fixation, which involves the binding of atmospheric nitrogen by cyanobacteria or bacteria such as Rhizobium, which live in symbiosis (mutualism) with the roots of leguminous plants [4]; (2) ammonification, a process involving the breakdown of organic nitrogen compounds through the decomposition by microorganisms (soil bacteria, fungi) of dead plants, animals, or manure into ammonia and ammonium ions [5,6]; (3) nitrification, a process carried out by nitrifying bacteria that oxidizes ammonia and ammonium salts into nitrites, and subsequently into nitrates, which in turn can be easily absorbed by plants [7]; (4) assimilation, a process in which plants take up NH_4_^+^ and NO_3_^−^ ions, which are then used by plants as building blocks for proteins, nucleic acids, or adenosine-5′-triphosphate (ATP) [8,9]; (5) denitrification, a process carried out by denitrifying bacteria, through which nitrates from the soil can be released into the atmosphere as N_2_ [10]. The biogeochemical cycle of nitrogen is shown in Figure 1. Furthermore, one of nitrogen’s most important roles is that it serves as a building block of life. As elemental nitrogen, it plays a vital role as a macronutrient; it is a component of amino acids, which in turn build proteins, as well as nucleotides (the nitrogenous base is one of the components, along with pentose and the phosphate group), from which nucleic acids are formed: deoxyribonucleic acid (DNA) and ribonucleic acid (RNA). It is also a component of chlorophyll, thereby enabling photosynthesis. In plants, nitrogen is taken up in the form of ammonium and nitrate ions, where it plays a key role in the growth of roots, leaves, stems, shoots, and fruits [11,12,13].

Nitrogen in the environment occurs primarily in the form of organic nitrogen and in the form of inorganic nitrogen, which is the most easily absorbed form of nitrogen by plants. The presence of inorganic forms of nitrogen, such as nitrates or ammonium, is crucial, but unfortunately, in excess, it can do more harm than good. Above-average levels of these compounds in surface water (rivers, lakes, seas) cause excessive growth of algae and cyanobacteria, leading to water eutrophication, reducing oxygen levels in the water, and consequently causing the death of aquatic animals [14,15,16,17,18,19]. Nitrates can also contaminate groundwater, thereby reducing the quality of drinking water. According to the World Health Organization (WHO), the nitrate content in water should not exceed 50 mg/L. Nitrate pollution in water is mainly caused by the runoff of NO_3_-fertilizers from fields into water bodies, leaky septic tanks, and pollutants’ emissions from industrial areas [18,19]. Meanwhile, regarding ammonium, the Polish Ministry of Health states that the limit for these ions is also 50 mg/L. Excessive ammonia levels in water are often an indicator of organic contamination, such as from sewage or water treatment issues, and high levels of NH_4_^+^ ions may suggest microbiological contamination [20]. In soil, an excess of ammonium ions leads to the release of hydrogen ions, soil acidification, and the leaching of other minerals, while an excess of NO_3_^−^ ions weakens plants, making them more susceptible to pests and fungal diseases [21].

**Figure 2:**
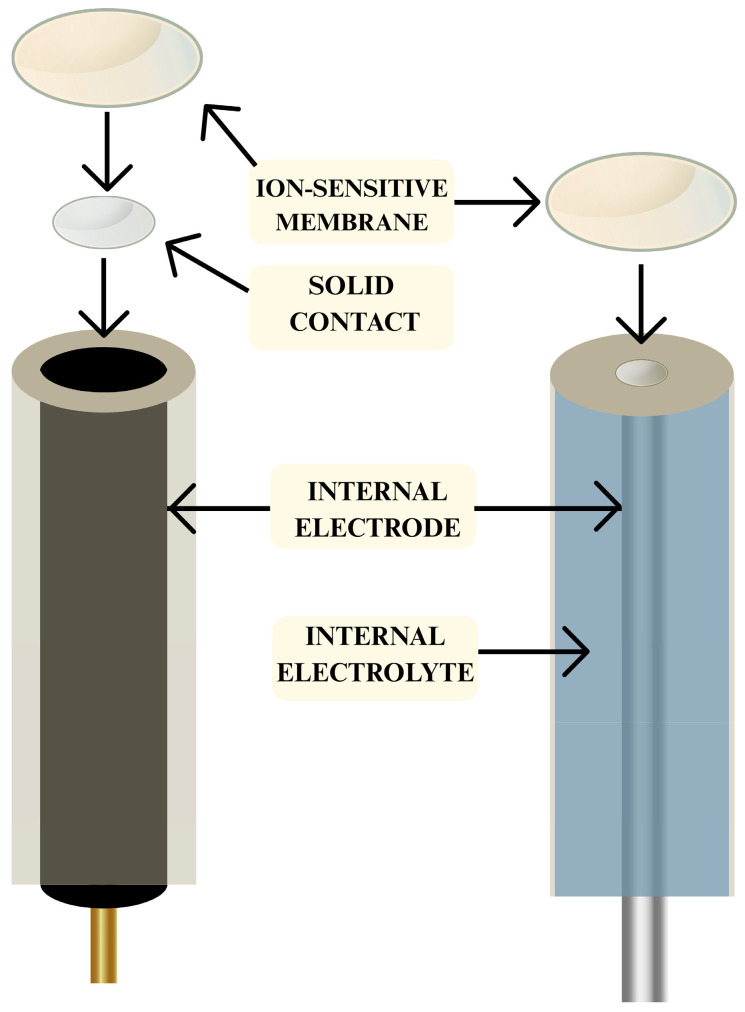
The construction of liquid contact and solid contact ISEs.

Based on the above, it is necessary to monitor aquatic ecosystems and soil for ammonium and nitrate ion levels. Research is being conducted in industry and at wastewater treatment plants, as well as in surface and groundwater; in agriculture, soil diagnostics are being carried out, which contributes to the development of intelligent and precise fertilization practices [22,23]. In addition, nitrate levels are monitored in fruits and vegetables [24], as well as in other foods where preservatives have been used, e.g., sodium nitrite in canned foods and cured meats [25]. In the case of ammonium, studies are conducted primarily in biological contexts, e.g., analysis of sweat and urine composition [26]. Classic methods used in the determination of mineral nitrogen include spectrophotometry, including the Nessler method for determining ammonium ions and the Griess method for determining nitrate (V), ion chromatography, fast flow analysis (FIA), and capillary electrophoresis [16,27]. These methods provide good results, but they have numerous limitations. They are quite expensive, and the analysis is not cost-effective, not only due to the price of the equipment used, but also because of other factors such as sample preparation time—which is often lengthy and requires sample purification—the duration of the measurement itself, and the use of expensive reagents that are frequently toxic and environmentally unfriendly. In addition, when using these methods, we cannot perform in-situ measurements. Therefore, an alternative solution is being sought, and here the problem of costly, time-consuming, and environmentally unfriendly analyses can be solved by using potentiometric sensors, namely ion-selective electrodes (ISE) [28,29,30]. The first ISE for pH measurement was developed as early as 1909 by Haber and Klemensiewicz [31]. ISEs are simple analytical tools that have been used in chemistry for decades; their operation relies on measuring the electromotive force of a cell comprising an ISE and a reference electrode (RE) [32]. They are mainly divided into liquid-contact (LCISE) and solid-contact (SCISE) electrodes, which consist of several components: an internal electrode, an ion-sensitive membrane (ISM), an internal electrolyte solution (IE) (in the case of liquid-contact electrodes (LC)), or an intermediate layer (solid contact (SC)), the so-called transducer medium (in the case of solid-contact electrodes). These media act as efficient ion-to-electron conductors, significantly enhancing charge transfer processes at the electrode interface. As a result, they contribute to improved electrochemical performance by ensuring a more stable, consistent, and reversible electrode potential over repeated cycles [29]. A diagram illustrating the appearance of the classic SC and LC electrode structures is shown in Figure 2.

**Figure 3:**
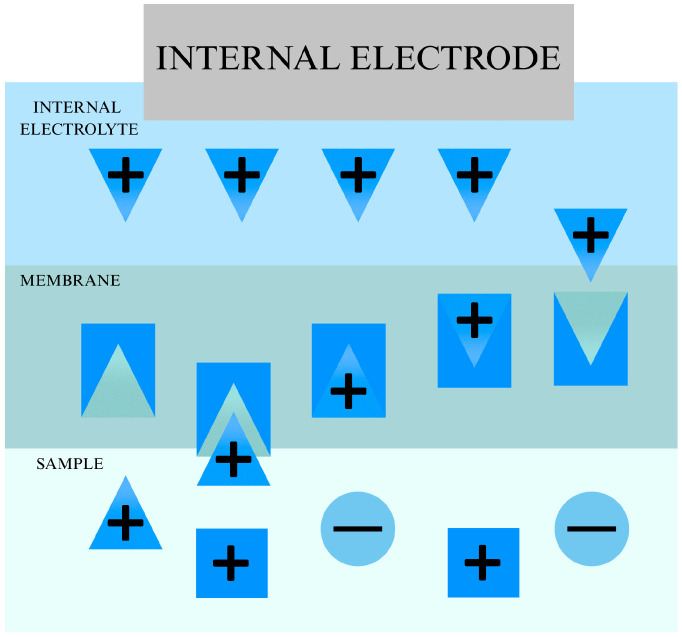
The mechanism of ionophore action.

The most important component of any ion-selective electrode is the ion-sensitive membrane, which provides selectivity for a specific ion even in the presence of interfering ions. In most cases, these membranes are made of polymers. Such an ISM consists of a sensitivity-providing element, the so-called ionophore or other active substance, a plasticizer that provides flexibility, a polymer serving as the polymer matrix (mainly polyvinyl chloride (PVC)), as well as an ion exchanger—usually salts containing large lipophilic ions. Of the elements listed above, the most important is the ionophore, which gives the electrode its selectivity. It is the ionophore that selectively reacts to a specific ion. The mechanism of action of the ionophore is shown in Figure 3.

**Figure 4:**
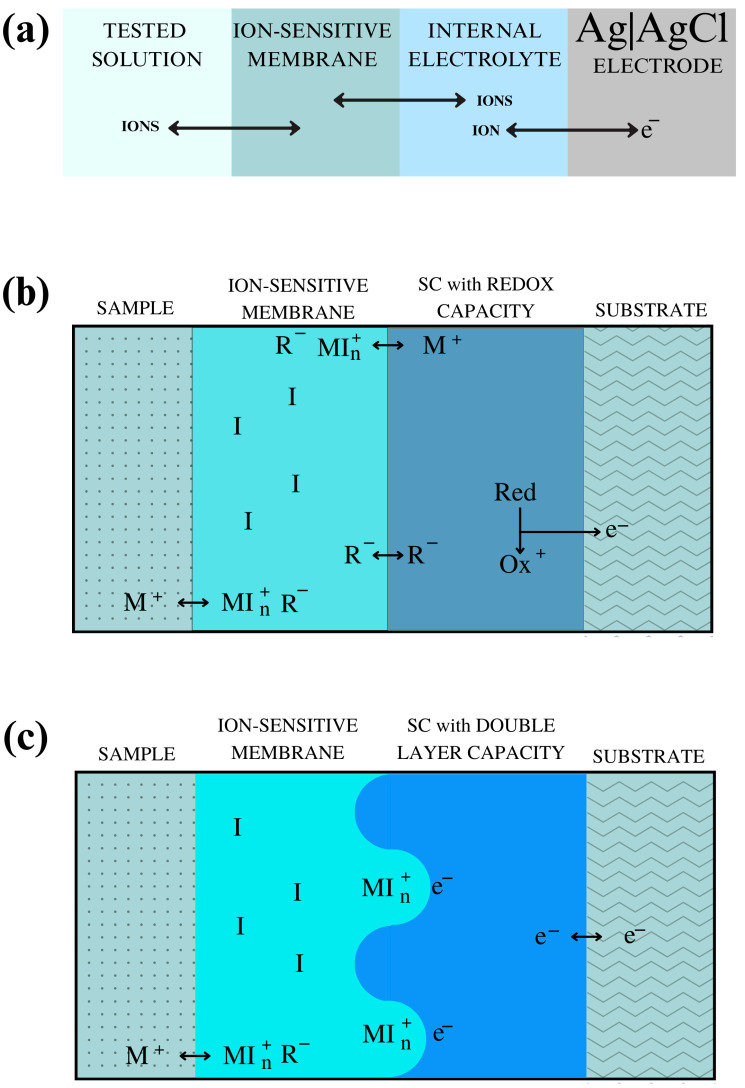
The mechanism of charge transport for liquid contact ion-selective electrode (**a**) and solid contact with redox capacity (**b**) and double layer capacity (**c**) ion-selective electrode (e^−^—electric charge; I—electrically neutral ionophore; M^+^—target ion, cation-sensitive membrane; R^−^—hydrophobic ion, anionic site, Red—reduced form of the molecule; Ox—oxidized form of the molecule).

The detection process involves a charge exchange that takes place between the sample solution, the membrane, the internal electrolyte or solid contact, and the internal electrode. Depending on the electrode design, this process occurs in different ways. The mechanism of charge transport for liquid and solid contact electrodes is shown in Figure 4.

Each of these electrodes has its advantages and disadvantages. Still, observing trends in science, there is a shift away from (LC) structures, and all-solid-state or solid-contact electrodes are primarily used. This is due, among other things, to the elimination of internal electrolyte from the design of solid-contact electrodes, making storage, transport, measurements, and sensor operation simpler [33]. The elimination of IE has paved the way for a new generation of sensors—enabling their miniaturization, the creation of custom shapes, and the development of screen-printed electrode (SPE) technology [34], and also enabled the use of electrodes in field studies or in more complex structures, such as wearable sensors [35], artificial tongues [36], and multi-electrode arrays for the simultaneous detection of several ions [37]. Unfortunately, these electrodes exhibit one major drawback—relatively poor ion-to-electron conductivity resulting from differences in conductivity at the membrane–internal electrode interface. The solution to this shortcoming is the application of a layer, component, or additive to the membrane that ensures good charge transfer between the sample, the membrane, and the electrode contact [38]. Current research focuses on various design approaches and modifications to develop sensors with enhanced stability, reversibility, repeatability, sensitivity, and selectivity.

This advancement also applies to ammonium and nitrate electrodes, which are the focus of our discussion. In ammonium-selective electrodes, nonactin is the most commonly used ionophore; however, these electrodes often exhibit some cross-sensitivity to potassium ions. Conversely, the problem with nitrate electrodes is the presence of chlorides, which can interfere with the operation of NO_3_^−^ ISEs. Many sensors have been developed to date, but in this paper, we will focus on the years 2020–2026. The paper collects, compiles, and describes 64 electrodes sensitive to nitrate (V) ions, along with 22 electrodes sensitive to ammonium ions.

## 2. Nitrate-Ion Selective Electrodes

Nitrate electrodes constitute a very broad class of potentiometric sensors. The literature describes a vast array of these sensors—ranging from solid-contact sensors to liquid-contact sensors, sensing platforms, and even commercially available sensors. In our review, we present a collection of 64 nitrate-selective electrodes developed between 2020 and 2026. These electrodes have been used to determine nitrate levels in soil and water samples, as well as for other types of measurements; alternatively, we present electrodes described solely in terms of their design and laboratory testing, without considering their practical applications. The electrodes are listed in a table and described in detail in the individual chapters. The table provides an overview of the electrodes’ construction—including the internal electrodes used, modifications to the buffer layers, and other features, as well as a summary of their analytical parameters: slope, linearity range, detection range, stability, and pH.

### 2.1. Determination of Nitrates in Soil

Over the past six years, 15 different sensors have been designed and subsequently used to determine nitrate (V) ions in soils. Almost all of them were solid-contact electrodes, except for one developed by the Nakao team [39]. Their work utilized an internal silver chloride electrode, which is standard for this type of setup, but it was modified from the Ag|AgCl|Cl^−^ configuration to Ag|Ag^+^. The internal electrolyte consists of 50 mM AgNO_3_ and 50 mM Mg(NO_3_)_2_. Tetraheptylammonium nitrate (THANO_3_) was used as the ionophore, forming a PVC-membrane (poly(vinyl) chloride) composition together with 2-nitrophenyl octyl ether (NPOE). Among the solid-contact electrodes, tridodecylmethylammonium nitrate (TDMANO_3_) played a significant role as an ionophore. It was used as a selectophore in three SCISEs. Two of these are based on a glassy carbon electrode, but different intermediate materials were used in each. A hybrid material consisting of a metal oxide (ZnO) and a noble metal (Pt, Au, and Ag) was used in the study [40]. The values obtained for the ZnO:Pt-modified electrode are described in Table 1. In contrast, the second research team used bimetallic selenium compounds (CoWSe_2_) as the transducer medium [41]. Both types of electrodes exhibited high sensitivity >61 mV/dec, as well as a detection limit in the micromolar range. The electrode modified with a hybrid material exhibited a wider linear range (LR) than the other one. Another study presenting the use of TDMANO_3_ involved a fully integrated potentiometric soil nitrate sensor array. This screen-printed sensor was used to monitor nitrate content in various soils cultivated with corn, cowpea, and tomato. Measurements of NO_3_^−^ were performed at different depths in two soil types: clay soil and sandy loam soil [42]. This electrode exhibited a super-Nernstian slope of −81.76 mV/dec, a relatively low limit of detection (LOD), but unfortunately, a rather narrow linearity range. Quaternary ammonium salt-based (TDA^+^) ionophores constitute a fairly large group of compounds used in NO_3_-ISEs—another example is the use of tetradodecylammonium bromide (TDABr) in a PVC membrane within a glassy carbon electrode (GCE) design, with polyaniline (PANI) serving as the transducer medium [43]. A relatively high sensitivity of −58.6 mV/dec was obtained, but with a rather large deviation of 5.2 mV/dec, indicating quite low repeatability and poor long-term stability. Electrode stability in this case was defined using a relative standard deviation (RSD) of 1.2%. Sometimes TDABr is replaced by tridodecylammonium chloride (TDACl). So it was with the electrodes proposed by Fayose et al. [44]. The anion is only an accompanying anion, and the actual active component is the TDA^+^ cation (quaternary ammonium). During conditioning, either Br^−^ or Cl^−^ ions are replaced by NO_3_^−^ ions anyway. Both of the presented electrodes were developed in a rather unconventional way—their substrate was applied to an acetate sheet of appropriate roughness by drawing a line with a B-grade pencil, and then the resulting line was covered with tape with a hole for the membrane (thus creating a matrix on which the RE, formed in the same manner, was also located). Sensitivity was not specified in any of the presented studies. Despite an LOD in the μM range, both of the presented electrodes exhibited a short linearity range. Another example of an ion exchanger is tetradodecylammonium nitrate (TDANO_3_), which was used in a screen-printed carbon electrode [45]. The study focused more on the determination of nitrates in various soil types than on analytical parameters—the screen-printed carbon electrode (SPCE) demonstrated measurement capabilities for NO_3_^−^ ion concentrations in the range of 5 to 512 ppm. In another case, an SPCE using conductive nano-C ink (JC81) as the transducer medium employed tetraoctylammonium bromide (TOABr) as the ionophore [46]. However, compared with other electrodes—such as those based on GCE—despite a high slope of -58 mV/dec, a narrower linearity range (1 × 10^−1^–1 × 10^−4^) was obtained, but with relatively good long-term stability of 0.03 μV/h. Another type of electrodes used for nitrate determination (this time in unfertilized, naturally fertilized, and chemically fertilized soil) are carbon paste electrodes (CPE) (based on carbon black (CB)) modified with hydrated iridium oxide (CB + IrO_2_·H_2_O) or hydrated ruthenium oxide and poly(3-octylthiophene-2,5-diyl) (CB + RuO_2_·H_2_O + POT) [47]. To achieve selectivity, nitrate ionophore V was used (the sensitivity of both electrodes was around 57 mV/dec, and their linearity range was five orders). The advantage of these SCISEs is undoubtedly their wide operating pH range. The other selectivity provider was the commercially available ionophore nitrate ionophore VI, which was used in the study by Baumbauer et al., where a gold screen-printed electrode was investigated [48]. In this case, a significantly weaker slope of 54.2 mV/dec was obtained with a linear range (LR) similar to that of other SPEs. However, this electrode exhibited poor repeatability as determined by the E^0^ parameter (E^0^ variation equal 12.5 mV). In addition to the aforementioned materials, PPy-NO_3_ is also used as an active membrane component. It was employed in electrodes coated with a composite of gold nanoparticles (AuNPs) and electrochemically reduced graphene oxide (ERGO), which served as an intermediate layer [49]. Ming et al. report that the electrode formed over 65 days achieved an average slope of 44.02 mV/dec, indicating low sensitivity. The electrode developed by the Chen research group exhibited a similar lifespan, though they do not report the sensitivity [49]. In addition to this type of electrode, a more complex system was developed, the so-called Cooperative Ion-Selective Electrode System, which, in addition to the ISE, incorporated a temperature and pH sensor into its design [50]. The sensitivity achieved here (51.63 mV/dec) is not groundbreaking, and the linearity range is narrow. However, it is worth noting that this type of design is a very good solution for in-situ measurements. In summary, the internal electrodes used included glassy carbon electrodes, paste electrodes, screen-printed substrates, and composite structures. In turn, compounds from the group of quaternary ammonium salts, i.e., TDMANO_3_, THANO_3_, TDANO_3_, TDACl, TDABr, TOABr, polymeric ion exchangers, i.e., PPy-NO_3_, as well as commercial ionophores, i.e., nitrate ionophores V and VI, were used as ionophores. The best solution turned out to be glassy carbon electrode structures modified with composites or carbon materials, which offer high stability, reproducibility, sensitivity, and a wide measurement range (both concentration and pH).

### 2.2. Determination of Nitrates in Water

According to World Health Organization guidelines and European Union regulations, the recommended maximum nitrate concentration in potable water is 50 mg/L. Therefore, monitoring these ions is essential, especially in drinking water. For this purpose, we can use potentiometric sensors. Table 2 provides an overview of 18 electrodes used in the analysis of various water samples. Fourteen of the presented electrodes are solid-contact ISEs, three are liquid-contact electrodes, and one is a commercial electrode. As with soil analysis, TDMANO_3_ is the most popular ionophore here as well, having been used in 7 sensors. Among all these SCISEs, the electrode presented by the team of Morawska et al. (60.41 mV/dec) not only exhibited the highest sensitivity but also the widest linearity range (10^−1^–10^−6^ M) [51]. In this case, a GCE coated with a composite of multi-walled carbon nanotubes (MWCNTs) and copper oxide nanoparticles (CuONPs) was used as the internal electrode. In the study [52], a similar design was presented; however, octadecyl amine-functionalized single-walled carbon nanotubes (f-SWCNTs) were used as the intermediate layer, and the analytical parameters were inferior to those of the aforementioned SCISE. Aside from GCE, a gold electrode modified with a POT and MoS_2_ nanocomposite was also used as the internal electrode [53]. In this case, the electrode in question exhibited lower sensitivity and a measurement range of only four orders of magnitude. Furthermore, it was characterized by a large potential shift. The LOD was determined in solutions already containing interfering ions—which is not a typical approach—and ranged between 3.23 × 10^−5^ and 1.65 × 10^−4^ M. Because of this, we were unable to directly compare these results with those reported for the other presented electrodes, due to the different method used for LOD determination. In addition to the use of the TDMANO_3_ ionophore on a macroelectrode scale, it is also used in membrane coating screen-printed electrodes, i.e., in the work by Darestani et al. [54], where the substrate was a hydroxylated glass slide with pencil contact pads and Cu contacts, which was then coated with a film of pristine CNT and a CNT–TPM. This is a rather interesting solution, but unfortunately, it does not yield satisfactory results in terms of electrode characterization. As the authors state in the summary of their work, a better solution for this type of electrode is to introduce cobalt(II) tert-butyl-salophen as the ionophore, which allows for maintaining a similar detection limit value, with the additional advantage of an expanded ISE operating range. A screen-printed electrode for the determination of nitrates in various types of water was also used by the research team of Thuy et al. [55]. In this case, the intermediate layer consisted not of a carbon material, as in Darestani et al. [54], but of cobalt oxide nanoparticles. This proved to be a much better solution, as a very low detection limit (1 × 10^−8^ M) was achieved (the lowest among electrodes using TDMANO_3_ as an ionophore). Thanks to the use of the intermediate layer, no water layer was observed. In addition to the above examples, TDMANO_3_ has been used in a polymer composite-modified paste electrode to improve the electrode’s electrical performance [56]. A drawback of this electrode is that after approximately 20 days, it significantly lost its sensitivity, with the slope value decreasing from −51.34 mV/dec to approximately −40 mV/dec. In turn, the team Hjort et al. presented a different solution—they developed a nitrate ISE based on hydrophobic laser-induced graphene (LIG) coated with a PVC membrane containing TDMANO_3_ [57]. Despite a relatively narrow linearity range, a response close to the Nernst equation was achieved, along with an LOD in the micromolar range. Unfortunately, this electrode, in turn, has a limited operating pH range, similar to a neutral environment (6–8). In another screen-printed design, a nitrate VI ionophore was already used—the authors modified the SPET electrode (eDAQ, ET083) with poly(tetrafluoroethylene) (PTFE) [58]. Apart from the SCISEs described above, many other designs differ significantly from one another. For example, Zhang et al. [59] propose modifying the GCE with gold nanoparticles (acting as a mediating layer) and then coating them with a membrane containing the ionophore PPy-NO_3_, whereas Pietrzak et al. [60] choose Ag|AgCl as the internal electrode, with the Co(Bphen)_2_(NO_3_)_2_(H_2_O)_2_ complex serving as the ionophore. Despite similar operating ranges, the team of Pietrzak [60] developed a more sensitive, more selective electrode capable of micro-level determinations. A rather interesting NO_3_-ISE solution was presented in the paper [61]. The copper wire was immersed in graphite-epoxy, and after drying the applied layer at 50 °C, a membrane containing 1,3,5-tri (p-hydroxyphenyl)benzene-based chlorotriazine pillared cage molecule (CAGE-1). A very good potentiometric response was obtained, although the working electrode undoubtedly requires improvement in sensitivity. The electrode nevertheless exhibited relatively good reversibility, selectivity, and a pH range suitable for the determination of nitrates in samples. Graphite-epoxy was also used in other electrodes, in part as a transducer medium and as a substrate, since it served as a base onto which a PVC membrane modified with 1-furoyl-3,3-diethylthiurea (a component ensuring selectivity for NO_3_^−^) was subsequently applied [62]. Membrane activation was performed in various solutions, which also provided an interesting comparison and a novelty in the studies described to date; for this purpose, solutions of Pb(NO)_3_, KNO_3_, and H_2_O, for which slopes of −65.2, −38, and −23 mV/dec, respectively, were obtained. Typically, simpler salts, such as KNO_3_, are used in conditioning solutions; this was employed, for example, by Morawska et al. [51]—in their case, a high slope was obtained without the need for toxic reagents, as the use of lead compounds is not environmentally friendly and generates unnecessary waste that is difficult to dispose of. Furthermore, the resulting electrode characteristics are weaker. The electrode was used in nontypical determination of chloride ions by potentiometric titration. A significantly better approach for screen-printed electrodes intended for the determination of NO_3_^−^ ions is the use of an alkyl ammonium salt—TDANO_3_—as the ionophore, as demonstrated by Gil et al. [63]. A commercially available CP electrode (ref. DRP-110) was modified with graphene oxide (GO) before ISM application. This resulted in good electrode response, as well as low potential drift (0.08 μV/s) and a very wide pH range (3–11). One of the more complex designs, known as ion-sensitive field-effect transistors (ISFETs), was described in a 2020 paper [64]. It is a large multisensing platform used to analyze aquarium water, sensitive to, among other things, nitrate (V). The manufacturing process involved first depositing a graphene layer onto a silica wafer, followed by a metal layer (in this case, a copper foil), which was then covered with a commercial membrane from CleanGrow. Given the complex structure of the sensing platform, very good measurement parameters were achieved, and the quality was confirmed through analytical applications. The remaining electrodes are based on an LC design—that is, one containing an internal solution acting as a charge-carrying medium/transistor. The research group of Cuartero et al. developed the so-called “electronic tongue” [36]. The ISE body was obtained from Fluke and filled with 1 × 10^−3^ M KNO_3_. The membrane utilized the TDMACl ionophore. Unfortunately, the paper did not include potentiometric or electrochemical results. The focus was on quantification. The same ionophore was used in a similar design, with the only difference being the IL (a mixture of KCl and KNO_3_ at a concentration of 1 × 10^−2^ M) [65]. Despite its near-Nernstian slope, the electrode had a fairly narrow measurement range. Furthermore, the stability determined from the calibration curve is not very accurate and cannot serve as a reference for other studies that include these data. The last type of ISE, or more precisely the IoT-Based Nitrate Measurement System, was described in the paper [66]. The working electrode was a silver chloride electrode filled with a 0.01 M NaNO_3_ and NaCl solution. The selectivity was provided by a TDANO_3_-membrane. To summarize the above information, as in Section 2.1, the most effective, accurate, stable, and sensitive instruments are those using GCE [51] and the TDMANO_3_ ionophore [63] or Co(Bphen)_2_(NO_3_)_2_(H_2_O)_2_ [60]. And once again, solid-contact electrodes offer us more possibilities than liquid-contact electrodes.

### 2.3. Other Analytical Applications of NO_3_-ISEs

Nitrates are not found only in water or soil, but also in other media or solid samples. Consequently, electrodes are being developed for various applications involving environmental, industrial, plant, and medical samples. This is why the development of various measurement tools is so important; these tools will not only streamline but also facilitate the work of analysts, while providing relatively reliable and reproducible results. Table 3 presents a summary of 15 electrodes, of which only one is an LC-type electrode. These designs often utilize a TDMACl membrane, which acts as an ion exchanger. This is also the case for the electrode used by Miras et al. to determine nitrates in nutrient solutions [68]. For this purpose, a commercial ISE (Sigma 45137-1EA) was used, filled with 1 × 10^−3^ M KCl. In the case of solid-contact electrodes, ammonium alkyl salts again dominate as ionophores. TDMANO_3_ was used in three electrodes. Two of these were GCE electrodes modified with polyaniline nanofibers doped with NO_3_^−^ (PANINFs-NO_3_) or Cl^−^ (PANINFs-Cl) ions [69]. Each of these electrodes achieved very good analytical performance, including an impressive linearity range (10^−1^–10^−6^) and a pH range of 4–11.5. The PANINFs-NO_3_ doped electrode exhibited better sensitivity, likely due to the presence of the dopant. The third electrode, based on TDMANO_3_, is a graphite SP electrode, which serves as a platform with RE (AgCl) [70]. Both electrodes are extremely small (1 mm), and the entire device is visually smaller than a 1-euro coin. It exhibited a good, though weaker, electrochemical response than GCE-SCISEs. The authors specify a pH range of 4 to 11; however, changes in electrode potential within this pH range are quite large (several to over a dozen millivolts), and significant discrepancies depend on the direction of the solution’s pH change (alkaline or acidic). Another ionophore from the group of alkyl ammonium salts is methyl tetradecyl dimethylamine nitrate (MTDANO_3_), which was used in a membrane deposited on chemically reduced graphene (CRGN), which was placed on a GCE [71]. The sensitivity of these electrodes is similar to that of the GCE-ISE presented by Pietrzak et al. However, it is worth highlighting an interesting application in the determination of NO_3_^−^ in PM2.5. In the case of another ion exchanger—TDANO_3_—it was used in three SC electrodes. Two of them are screen-printed electrodes with a carbon substrate. One of them was enhanced with an intermediate layer of reduced graphene oxide aerogel (rGOA) [72], and the other with poly(3,4-ethylenedioxythiophene)-polyethylene glycol (PEDOT:PEG) [73]. Taking into account that, in most cases, SPEs exhibit a poorer response than, for example, glassy carbon electrodes, the Kim team has developed a groundbreaking electrode [72]. It features extremely high sensitivity, which additionally spans a wide linearity range, resulting in a low LOD (7.59 × 10^−7^ M). This electrode, when compared with the one developed a year later by Hao et al., performs significantly better. The third SCISE presented in this mini-review is a micro-sized all-solid-state electrode, where the reference electrode is a copper wire, and graphite serves as the transducer medium [74]. This electrode exhibits a typical response for a NO_3_-ISE. Unfortunately, its stability is defined only as “high” due to the slope remaining at high values. In addition to the aforementioned ammonium ion exchangers, TOANO_3_ was also used. It was employed in an “alternative membrane”—photocurable poly-tetrahydrofurfuryl acrylate (pTHFA) [75]. This is a rather interesting design among screen-printed electrodes, as this type of membrane has not been seen before. This membrane was applied to a PPy conductive layer previously applied to a commercial single-strip screen-printed electrode (S3PE). Despite its groundbreaking design, the results were not spectacular—average sensitivity, as well as a fairly high detection limit and linearity range. It was used to determine nitrate(V) ions in fish ponds, river water, and soil. Another type of ion exchanger involves Br^−^ anions, such as TDABr or TOABr. TDABr was incorporated into a PVC membrane, which was applied to a solid-contact chitosan and black phosphorus combined with ferric oxide (IL to GCE) [76]. TOABr, on the other hand, was part of an ISM applied directly to a DuPont 7102 carbon paste printed electrode [77]. Considering the research results presented in the publications, the GCE-type electrode outperforms the screen-printed ISE in terms of parameter values. Moving on, typical nitrate VI and V ionophores were used, respectively, in electrodes for measurements in interesting samples—blood [78] or special plant substrates [79]. The first of these electrodes is a gold electrode coated with a transducing layer in the form of thiol-functionalized reduced graphene oxide (TRGO) [78]. This type of modification resulted in a two-week longer electrode lifespan compared with the use of ordinary rGO. In the case of this ISE, special attention should be paid to the wide pH range, which allows for a broad spectrum of applications. The second electrode mentioned is a paste electrode, which is a combination of carbon black and hydrogenated ruthenium dioxide [79]. Even though the slope is lower, this electrode has the same pH range, a similar LOD as above, and an even wider linearity range. The advantage of this electrode is its high stability—the potential drift is only 0.19 mV/h. The last electrode in this section of the review is a GCE-based ISE, which this time has been enhanced with a conductive layer in the form of pericarpium granati-derived biochar with phosphoric acid activation (PGCP), which was coated with polypyrrole (PPy) to form a so-called bilayer membrane nitrate ion-selective electrode [80]. Compared with the previously described electrode, it exhibits similar properties but a narrower pH range and poorer stability. To summarize this section, we can once again clearly state that GCE-based electrodes are superior in terms of quality. They provide reproducible results, good long-term stability, are simple and inexpensive to prepare, and do not require complex designs.

### 2.4. NO_3_-ISEs Without Analytical Application

Once new sensors are developed, they are not immediately used for analytical determinations. Many studies present new solutions in the field of potentiometric sensors. Over the past 5 years, 16 types of such ISEs have been developed—the information about these electrodes is placed in Table 4. As in the previous subsections, we will begin our discussion with electrodes in which compounds from the alkylamine group were used as ion exchangers in the ISM, and once again, TDMANO_3_ is the most popular choice in this case. It was used in a series of electrodes, each of which had a different internal electrode. The GCE-based design was modified by applying a conductive polymer layer of PEDOT-C14 [82]. Compared with other available NO_3_-ISEs, this is not a standout sensor. A significantly better solution is to introduce a conductive material in the form of commercially available purified mesoporous carbon (MCB) between the silver electrode and the membrane [83]. Higher sensitivity was achieved here, with an LOD in the micromolar range. In terms of the stability of this electrode, the drift in the first 5 min was 0.8 ± 0.6 mV (on a daily scale and in relation to long-term drift, it was defined as a range of 2–10 mV/day). The screen-printed design was also popular. The paper [53] presents a comparison of two electrodes of this type. In the first case, a nanocomposite of poly(3-octylthiophene-2,5-diyl) polymer combined with MoS_2_ was used as the transducer medium, forming an intermediate layer between the membrane and the Au layer. The second of the studied electrodes was a graphite SPE, where PPy was used as the transport/transistor medium. The PPy electrode exhibited higher sensitivity (lasting for approximately 30 days) and stability. In the carbon electrodes screen-printed at ISM, other ion exchangers from the group of alkyl ammonium compounds were also used, namely TDANO_3_ [84] and TOANO_3_ [85]. In the first case, an additional modifier was used, which ensured better ion-to-electron conductivity in the poly(vinyl acetate) PVAc@NaNO_3_ electrolyte layer. As for SPE, very good sensitivity and stability of 0.73 μV/s were obtained. In the second case, however, the parameters were poorer; however, it should be noted that Goodrich et al. developed not a single electrode, but fully printed ion sensor arrays for measuring agricultural nitrogen and potassium. They presented an interesting approach using artificial intelligence (AI). Four of the SCISEs presented in this subsection utilized the nitrate ionophore V. In the paper [86], a platinum electrode was used, onto which an ion-to-electron transducer layer of manganese dioxide and poly(allylamine) (PAAm-MnO_2_) was applied. Despite the low sensitivity, quite good LOD and linearity ranges were achieved. Three years later, sensors with higher sensitivity and very good stability were developed [87]. In this case, the GCE electrodes were modified with carbon materials: graphene, carbon black, and carbon nanotubes. The use of graphene proved to be the best solution. In turn, nitrate ionophore VI was used in an ISM with a rather interesting design. A gold-coated plastic interdigitated working electrode was additionally coated with PPy, followed by PVC-ISM [88]. PPy-NO_3_ was also used as the ion exchanger. It was used in two studies—both research teams utilized a GCE onto which Ni-HAB MOF (high-capacity metal-organic framework) was deposited as an ion-to-electron transducer [89], or a gold layer deposited via Au layer-by-polymerization [90]. Both electrodes exhibited very similar characteristics, but the team led by Abdollahzadeh et al. [89]. reliably demonstrated potential drift, which was extremely low—1.3×10^−4^ μV/s—while the Gao team defined SCISE stability based on the slope [90]. In the case of GCE, a much more effective modification turned out to be the introduction into the ISM of a selectivity provider in the form of the Co(Bphen)_2_(NO_3_)_2_(H_2_O)_2_ or a nanocomposite of carbon material and ionic liquid (MWCNTs-THTDPCl). This solution ensured not only a very good slope of over 57 mV/dec, but also one of the lowest LODs among all the electrodes presented in this review. An additional advantage is the absence of a water layer, as well as a fairly wide pH range. The carbon paste electrode, which utilized an additive in the form of dry battery residues, exhibited a similar response. This is an extremely eco-friendly solution that contributes to the development of “waste management” and can be used for the disposal of battery residues. However, further work is needed on the linearity range [91]. Another carbon paste electrode, which is the last in this comparison, has a substrate consisting of a mixture of carbon paste and dipropylene glycol dimethyl ether polyethylene terephthalate (PET). It is additionally plasma-treated but exhibits poor sensitivity (−48 mV/dec), a narrow linearity range, and is one of the less promising SCISEs [92]. In summary, here too, the modification of glassy carbon electrodes is the most effective, economical, and efficient approach. Often, highly complex designs have a relatively short lifespan and require further optimization or development to achieve the desired results that could contribute to the sensor’s commercialization.

### 2.5. Summary of Studies on Nitrate Ion-Selective Electrodes

This chapter describes 64 electrodes, with the vast majority being solid-contact electrodes, which are more universal sensors. To enhance the sensitivity of the potentiometric sensors, polymer membranes were primarily used. For nitrate electrodes, the main types of ionophores are alkyl amine salts, which act as ion exchangers—TDMANO_3_ (the most popular), TDANO_3_, TDMACl, TDACl, TDABr, TOABr, as well as other compounds such as PPy-NO_3_, or commercially available ionophores—nitrate ionophores V and VI. In addition, the intermediate layers (in the case of solid-contact electrodes) were modified—not only carbon-based, but also polymeric, composite, and hybrid materials were used. Among these electrodes, those with a glassy carbon substrate were most frequently used, as well as electrodes based on screen-printing technology. The most effective solution discussed in these subsections turned out to be the use of a GCE-based electrode, which was subsequently modified in various ways with conductive layers—ranging from carbon materials, through polymers and nanoparticles, to popular composite materials. The use of composite materials, as opposed to their individual components, yielded significantly better results in terms of both performance (high sensitivity and low detection limit were achieved) and electrode potential stability. Each of the presented electrodes is unique in its own way, and 48 of them were used for practical applications.

## 3. Ammonium-Ion Selective Electrodes

Compared with nitrate-selective ISEs, significantly fewer electrodes sensitive to ammonium ions have been reported. In our review of studies published between 2020 and 2026, we identified 22 different sensors, all of which were of the solid-contact type, with a large proportion consisting of screen-printed electrodes. The most commonly used ionophore is nonactin. This chapter has been divided into four subsections based on their applications.

### 3.1. Determination of Ammonium in Soil

Determining ammonium ion levels in soil is very important. The toxic concentration of NH4 ions for plants varies and depends on the type and species of plant. Studies report a wide range of values for the ammonium ion concentration tolerated by plants, from 0.75 mM to 100 mg/L [94]. When such high concentrations persist for a very long time, they negatively affect plant growth and harm humans and animals. Monitoring itself not only serves to determine the suitability of the soil for cultivation but can also contribute to the development of a smart crop fertilization system. Therefore, the contribution of science to the development of simple, inexpensive measurement tools is essential. Table 5 presents five different electrodes used for the determination of NH_4_^+^ ions in soils. All feature an ISM with nonactin as the ionophore. Morawska et al. proposed a GCE-type electrode in which a carbon nanocomposite (MWCNTs + CNFs) was used as the transducer medium [95]. This group of electrodes achieved the highest sensitivity (58.4 mV/dec), the lowest detection limit, and the widest linearity range. The electrode’s stability was also quite good, and the SCISE showed no sensitivity to external conditions, i.e., light or the presence of gases, and had a fairly wide pH range. The next electrode has the structure already described in Section 2.1, based on an acetate sheet on which a graphite line was drawn; however, there is no description of the potentiometric properties of the proposed ISE, as the study focused on the determination of mineral nitrogen in soil [44]. Nonactin was also used in three different screen-printed electrodes—one of them had a carbon substrate [96], and the other two had a gold substrate [97,98]. Despite its fairly good stability, the carbon-printed electrode exhibited rather low sensitivity, deviating significantly from the theoretical value defined for ISEs sensitive to monovalent ions. This may be due to the fact that the ISM was additionally coated with a layer of polyacrylamide hydrogel. The next two SPEs had gold substrates. Unfortunately, the paper [97] does not provide the basic parameters describing the SCISE, and it is difficult to make a comparison based solely on the linearity range, which is de facto narrower than that in the paper [98]. The most sensitive electrode and the one best suited for measurements in real samples turned out to be the one presented by the team of Morawska et al., so once again, and in the case of the NH_4_-ISE, this confirms that the GCE is a very good type of internal electrode.

### 3.2. Determination of Ammonium in Water

As with soil, there are certain restrictions on ammonium levels in water—the maximum concentration of ammonium ions in drinking water is 0.5 mg/L (2.77 × 10^−5^ M). The concentration of this ion in groundwater and other water sources is strictly dependent on the environment, pollution, and the degree of soil fertilization. Therefore, environmental monitoring, including for NH_4_^+^ ion concentrations, is essential. Table 6 lists six sensors used for determining ammonium ions in water samples. All of them are solid-contact electrodes of various designs. In all cases presented in this subsection, nonactin was used as the ionophore. In ISEs where the internal electrode was glassy carbon, AgNPs@MXene [99] or the gold nanoparticle-reduced graphene oxide nanocomposite [100]. Despite lower sensitivity in the case of the first of the mentioned SCISEs, it was possible not only to lower the detection limit but also to broaden the linearity range compared with the work by Nguyen et al. published two years earlier [101]. In the case of the AgNPs@MXene-modified electrode, high sensitivity of the potential to pH changes was observed (a potential jump between −3 and 197 mV), which is undoubtedly a drawback for the analysis of real samples, as it is necessary to adjust and maintain the pH at a relatively constant level, since the electrode exhibits large potential changes when the pH changes. Another example is a screen-printed electrode created by applying two strips of copper tape to CNT-TPM, onto which ISM was then applied with nonactin as an ionophore [54]. A competing electrode is another SPE developed by the team of Kamel et al. [102]. The electrode substrate was also a copper-based conductive ink modified with a PEDOT:PSS and MWCNTs composite before membrane application. This electrode was used for in situ measurements in an aquarium containing living organisms, thus involving a large number of potential interfering factors. It exhibited near-theoretical sensitivity (59.2 mV/dec), as well as a higher LOD compared with the SPE presented in the article [54], and a narrower pH range (though sufficient for the studies conducted in the aforementioned aquatic environment). The next two electrodes were also SPEs, but with a silver substrate applied from ink. In the first, the role of the ion-to-electron transducer medium was played by a rather interesting carbon-polymer composite (graphite particles and polyvinyl butyral) [103], while the second Ag/SPE was modified by screen-printing melamine-intercalated graphene nanosheets from ink [104]. Both of the presented electrodes exhibited very good analytical parameters, and, as noted by the authors Ivanisević et al., their electrode demonstrated exceptionally high stability. In the comparison presented in this section, screen-printed electrodes and modified GCE electrodes performed at a relatively similar level; however, the GCE/AgNPs@MXene electrode is particularly noteworthy, as it achieved a very low detection limit.

### 3.3. Other Analytical Applications of NH_4_-ISEs

The applications of NH_4_-ISEs extend beyond analyses conducted in soil or water. Ammonium sensors have been used in wearable sensor platforms to monitor ammonium ion levels in sweat. Moreover, they have also been employed for analyses in biological samples, such as urine. In this subsection, Table 7 presents a summary of the most important parameters for a total of 8 ion-selective electrodes with solid contact in which nonactin was used as the ionophore. The first SCISE of this section appeared in the literature in 2021—an Au electrode layer was deposited on a poly(ethylene terephthalate) (PET) substrate, which was then coated with PEDOT via electrodeposition. This electrode was only part of an entire electrode array used for the simultaneous detection of several ions in fresh crude vegetable leaf juices [105]. For such a design, a very high slope was achieved, indicating high sensitivity to NH_4_^+^ ions; however, this electrode suffers from a potential drift of 2–3 mV/hour. A much better solution using this ionophore was proposed by Niemiec et al. by creating a carbon black- and ruthenium hydroxide-modified paste electrode, which was used to determine ammonium ions in plant substrates [79]. They achieved similar sensitivity but approximately 13-fold better stability and a wider linearity range. Furthermore, an LOD in the micromolar range and a wide pH range. In subsequent GCE-based sensors, various intermediate layers were used, including stannic oxide (SnO_2_), titanium oxide (TiO_2_), and manganese oxide (MnO_2_) (since SnO_2_ yielded the best results, the data for this electrode are included in the table) [106] and a three-component composite of chitosan and black phosphorus combined with ferric oxide magnetic nanoparticles [76]. Thuy et al. achieved a very low detection limit of 10^−8^ M, which is the best result among all ammonium electrodes presented in this review; however, they were unable to maintain the electrode’s sensitivity, which was only 47.17 mV/dec. The type of oxide used did not affect the pH range of the electrodes. A noticeable difference was observed in the aqueous layer test, where only tin oxide yielded promising results (according to the authors, due to its layer hydrophobicity). Duan et al., in turn, achieved a sensitivity 10 mV/dec higher, and the electrode exhibited significantly better stability, confirming that the use of a solid contact material in the oxide:carbon composite is promising and warrants further exploration in this area. In the determination of ammonium ions in urine, an interesting design was used involving the deposition of graphene using a CO_2_ laser, which was then coated with a nonactin-based membrane. The electrode exhibited average performance, but it was successfully applied in the determinations [107]. In turn, screen-printed sensors with different substrates were used to monitor the concentration of NH_4_^+^ ions in sweat. In the first case, gold nanoparticle ink (working electrode) was used, along with a CNT-doped membrane that served as both an ion-to-electron transducer layer and an ion-sensitive structure [108]. The second example is an electrode consisting of a carbon ink layer, with MWCNTs-COOH serving as the transducer medium. It was used to analyze sweat from fingertips [109]. However, compared with the two electrodes mentioned above, the best one was proposed by the research team of Hua [110]. Their sensor consists of carbon and Ag/AgCl layers on a PU-PDMS substrate, which was enriched with graphene–carbon nanotube (Gr-CNT) 3D nanomaterials. Such a sensor, already equipped with a membrane (using nonactin as an ionophore), not only exhibited nearly Nernstian response, but also the widest linearity range among the screen-printed sensors in this subsection, as well as a promising detection limit. Furthermore, it exhibited high stability. Based on the analysis of these sensors, the modified GCE [76], the paste electrode [79], and the screen-printed sensor [110] would take the top three spots in this case.

### 3.4. NH_4_-ISEs Without Analytical Applications

The culmination of the discussion on ammonium electrodes is a compilation of those that have not been used or tested in practical ammonium determination. Only three of the 22 electrodes were not used for analytical determinations. The detailed information about these ISEs is presented in Table 8. Two of the SCISEs presented here feature a GCE as the internal electrode, while the third design is quite unusual. The GCE electrodes were modified either with a single layer of copper(II)-hexacyanoferrate (CuHCF) (i.e., the so-called ISM-free design) or with the same layer additionally coated with a nonactin membrane [111]. Despite the higher sensitivity achieved for the GC/ISM-free ISE—which is quite surprising—the linearity range and the lower limit of detection were reduced compared with the ISE with an ion-sensitive membrane. Furthermore, the ISM-free electrode exhibited poorer selectivity (except for the K^+^ ion) compared with the GC/CuHCF/ISM electrode. However, it is worth noting that the membrane electrode exhibited drift upon solution change, which was not observed for the ISM-free ISE. Both electrodes exhibited instability under varying light conditions. The third electrode mentioned in this comparison is the unusual and visually interesting SCISE. The working electrode consists of glass fibers coated with a special layer—radially aligned carbon nanotubes (RACNT) directly grown on glass fibers (GF) via chemical vapor deposition method [112]. This electrode demonstrated good sensitivity, linearity, and LOD; however, the designs of the previously mentioned electrodes are simpler, more user-friendly, and more cost-effective.

### 3.5. Summary of Studies on Ammonium Ion-Selective Electrodes

The ammonium electrodes described in the above subsections comprise a group of 22 sensors. Nonactin was primarily used as the ionophore in NH_4_ ISE electrodes, which facilitated the comparison of these sensors but also highlights the very limited availability of such ionophores and the need to develop new compounds that interact selectively with ammonium ions. All electrodes are solid-contact electrodes, and, as with nitrate-selective electrodes, glassy carbon and screen-printed electrodes dominate as substrates. The best results, characterized by high sensitivity, were obtained using composite materials as solid contact—both for GCE and SPE substrates. For these electrodes, nearly Nernstian slope values were obtained, as well as good potential stability. Looking toward further electrode development, it is worth focusing on new composite materials that could provide comparable or even better performance for potentiometric sensors.

## 4. Conclusions

Environmental monitoring to protect the environment and the development of eco-friendly analytical tools are crucial in today’s era of rapid technological advancement. Due to the high risk of water, soil, or other environmental contamination caused by excessive use—primarily in heavily industrialized areas, as well as agricultural regions where vast amounts of fertilizers are applied—it is essential to monitor the concentration of increased quantities of ion compounds that are toxic. One of the best solutions in environmental measurements is the use of inexpensive and simple potentiometric sensors. For nitrate and ammonium ions, 64 and 22 sensors, respectively, have been collected over the past six years. This large number is due to the demand for this type of measurement tool. The vast majority of the electrodes listed in the table have been successfully used to analyze various types of samples—water, soil, biological samples, and plant-based materials. Among the ISEs presented, those with solid contact dominated, and GCE electrodes proved to be the most effective, simplest, and most stable analytical tool. Similar conclusions can be drawn for electrodes sensitive to ammonium ions. GCE- based electrodes stand out at the forefront, along with certain SPCE-type electrodes—offering high sensitivity, low LOD, and good stability. Often, highly complex designs yielded significantly worse results or results very similar to those of simple solutions—complex, labor-intensive projects are not always the best solution. In summary, the main trends in the development of both sensor types primarily concern solid-contact designs. In this area, the majority of research is focused on the development and implementation of new types of electroactive materials in sensor designs, which can ensure high stability of sensor readings over both the short and long term. The analysis of the presented results suggests that composite and hybrid materials exhibit the highest potential for application. Because they are obtained by combining components with different properties, it is possible to design a material with targeted parameters. In this case, the most important factors are high electrical capacitance and high hydrophobicity. These parameters are crucial for producing sensors with stable potential, thereby eliminating the need for periodic calibration. The current trend in the development of potentiometric sensors—including those selective for nitrate and ammonium ions—certainly involves the continued development of miniature sensors using screen printing and 3D printing technologies, which enable the production of low-cost, often disposable sensors integrated with a reference electrode. The miniaturization of sensors aligns well with another research trend in ion-selective electrodes, which focuses on the design and development of multi-sensor platforms for the simultaneous analysis of multiple ions. The use of such platforms will allow for the acquisition of comprehensive information about a sample while simultaneously assessing the impact of interfering ions, such as Cl^−^ ions during nitrate determination or K^+^ ions during ammonium ion analysis. Taking into account that the main areas of application for nitrate and ammonium sensors include the analysis of natural waters and soils, we believe that the development of these sensors will focus on creating low-cost, durable sensors with high measurement stability for real-time and continuous monitoring of samples. This is facilitated by current and future advances in the design of sensors with constant contact and the now-advanced capabilities of remote signal transmission.

## Figures and Tables

**Table 1:** The ISEs used for nitrate detection in soil samples.

No.	Electrode Type	Working Electrode		Transducer Media	Selectivity Provider	Slope	Limit of Detection	Linearity Range	Stability	pH	Reference
1	SC	GCE		CoWSe_2_	TDMANO_3_	−61.9 ± 0.4	1 × 10^−6^	1 × 10^−6^–7.5 × 10^−2^	2.3 ± 0.4 µV/h	-	[41]
2	SC	GCE		ZnO:Pt	TDMANO_3_	−62.52	3.2 × 10^−6^	1 × 10^−1^–1 × 10^−6^	0.78 µV/s	3–10	[40]
3	SC/SPE	Carbon ink		POT:MoS_2_	TDMANO_3_	−81.76	1.05 × 10^−5^	1.45 × 10^−3^–7.45 × 10^−5^	-	-	[42]
4	SC	GCE		PANI	TDABr	−58.6 ± 5.2	1.67 × 10^−6^	1 × 10^−1^–1 × 10^−5^	RSD = 1.2%	3.5–10	[43]
5	SC	Graphite line		TDACl	-	2.14 × 10^−6^	0–1.43 × 10^−3^		-	-	[44]
6	SC	Graphite line		TDACl		3.1 × 10^−6^	0–3.23 × 10^−4^		-	-	[44]
7	SC/SPE	SPCE		-	TDANO_3_	-	-	5–512 ppm	-	-	[45]
8	SC/SPE	SPCE		Conductive nano-C ink (JC81)	TOABr	−58	<10^−6^	10^−9^–2.7	-	-	[46]
9	SC	CPE		CB + IrO_2_·H_2_O	Nitrate ionophore V	−57.2 ± 0.2	10^−5.22±0.05^	1 × 10^−1^–1 × 10^−5^	181 µV/s (I = 10 nA)	2–10	[47]
10	SC	CPE		CB + RuO_2_·2H_2_O + POT	Nitrate ionophore V	−56.9 ± 0.1	10^−5.15±0.05^	1 × 10^−1^–1 × 10^−5^	116 µV/s (I = 10 nA)	2–10	[47]
11	SC/SPE	Printed AuE		-	Nitrate ionophore VI	−54.1 ± 2.1	-	5 × 10^−5^–1 × 10^−1^	*E*^0^ variation was found to be 12.5 mV	-	[48]
12	SC	GCE		AuNPs + ERGO	PPy-NO_3_	-	10^−5.2±0.1^	10^−1^–10^−5^	-	-	[49]
13	SC	-	AuNPs + ERGO	PPy-NO_3_	44.02	-	-		-	-	[49]
14	SC	Cooperative Ion-Selective Electrode System			−51.63	8.06 × 10^−6^	10^−5^–10^−2.2^		-	-	[50]
15	LC	modified inner electrode system from Ag|AgCl|Cl– to Ag|Ag+.	50 mM AgNO_3_ and 50 mM Mg(NO_3_)_2_	THANO_3_	−53 ± 1	10^−5^	10^−5^–2		-	-	[39]

**Table 2:** NO_3_-ISE used for nitrate detection in aqueous samples.

No.	Electrode Type	Working Electrode	Transducer Media	Selectivity Provider	Slope	Limit of Detection	Linearity Range	Stability	pH	Reference
1	SC	GCE	(f-SWCNTs)	TDMANO_3_	−56	-	10^−1^–5 × 10^−6^	decreasing drift 0.4–0 mV/min	-	[52]
2	SC	GCE	MWCNTs + CuONPs	TDMANO_3_	60.41	5.13 × 10^−7^	10^−1^–10^−6^ 5.13 × 10^−7^	0.085 µV/s	-	[51]
3	SC	AuE	POT-MoS_2_	TDMANO_3_	55.9 ± 0.4	(3.23 × 10^−5^–1.65 × 10^−4^) ^a^	10^−1^–10^−4^	-	-	[53]
4	SC	pencil contact pads and Cu contact	CNT and CNT–TPM	TDMANO_3_	-	4.8 × 10^−6^	3.6 × 10^−5^–3.6 × 10^−3^	-	4–8	[54]
5	SC/SPE	SPCE	Co_3_O_4_NPs	TDMANO_3_	−56.8	1.04 × 10^−8^	10^−7^–10^−2^	-	3–8	[55]
6	SC/SPE	CPE	Polymer composite	TDMANO_3_	−51.34 ± 2.1 (after 20 days–40)	0.19log_10_(M)	10^−1^–10^−5^	Less than 10% drift over a month	-	[56]
7	SC	-	LIG	TDMANO_3_	−58.2 ± 4.2	6.01 × 10^−6^	5 × 10^−4^–1 × 10^−1^	-	6–8	[57]
8	SC/SPE	SPE (eDAQ, ET083)	PTFE	Nitrate ionophore VI	−58.03 (after 20 days–35)	-	10^−0.25^–10^−1.75^	*-*	*-*	[58]
9	SC	GCE	AuNPs	PPy-NO_3_	−50.4	5.25 × 10^−5^	5.25 × 10^−5^–1 × 10^−1^	-	-	[59]
10	SC	Ag|AgCl	Ag/AgCl/Cl^−^	Co(Bphen)_2_(NO_3_)_2_(H_2_O)_2_	−56.3	3.98 × 10^−6^	1 × 10^−5^–1 × 10^−1^	-	5.4–10.6	[60]
11	SC	Cu wire	Graphite-epoxy	CAGE-1	−50.3	7.5 × 10^−6^	10^−1^–10^−5^	-	4–9	[61]
12	SC/SPE	Graphite-epoxy		1-furoyl-3,3-diethylthiurea	−65.2 ± 0.7 (activation in Pb(NO_3_)_2_) −38 ± 1 (activation in KNO_3_) −23 ± 1 (activation in H_2_O)	24 ± 6.0 × 10^−6^	10^−2^–10^−4^ 24 ± 6.0 × 10^−6^	-	4–10	[62]
13	SC/SPE	SPCE (ref. DRP-110)	GO	alkyl ammonium salt	−53.5 ± 2.0	1.9 × 10^−6^	3.0 × 10^−6^–10^−2^	0.3 mV/h	3–11	[63]
14	SC/ISFETs	Cu foil	graphene FETs	Commercial membrane cocktails (CleanGrow)	56.7 ± 0.2	10^−5^	10^−1^–10^−5^	-	-	[64]
15	LC/electronic tongue	ISE body (Fluka)	1 × 10^−3^ M KCl	TDMACl	-	-	-	-	-	[36]
16	LC	ISE body	0.01 M KCl and 0.01 M KNO_3_ M	TDMACl	−59	9.3 × 10^−3^–5 × 10^−6^	-	-	-	[65]
17	LC/IoT-Based Nitrate Measurement System	Ag/AgCl	0.01 M NaNO_3_ + 0.01 M NaCl	TDANO_3_	-	-	-	-	-	[66]
18	Commercial ISE	LAQUAtwin ion selective electrode from Horiba, Kyoto, Japan			-	0.8 mg/L	0.8–90 mg/L	-	-	[67]

^a^ LOD was determined in the presence of interfering ions.

**Table 3:** The electrodes used to determination of nitrate ions in various samples.

No.	Electrode Type	Working Electrode	Transducer Media	Selectivity Provider	Slope	LOD	Linearity Range	Stability	pH	Sample	Reference
1	SC	GCE	PANINFs-Cl	TDMANO_3_	−56.8	3.15 × 10^−7^	10^−6^–10^−1^		4–12.5	Environmental samples	[69]
2	SC	GCE	PANINFs-NO_3_	TDMANO_3_	−57.8	1.12 × 10^−6^	10^−6^–10^−1^		4–11.5	Environmental samples	
3	SC/SPE	GrE	graphite	TDMANO_3_	−55.4 ± 0.7	2.04 × 10^−4^	2.9 × 10^−4^–1.7 × 10^−1^		4.0–11.0	Industrial	[70]
4	SC	GCE	CRGN	(MTDANO_3_)	−57.8	25 × 10^−6^	0.1 mM–0.1 M	-	-	PM2.5	[71]
5	SC/SPE	SPCE	rGOA	TDANO_3_	−59.1	7.59 × 10^−7^	1 × 10^−6^–1 × 10^−1^		-	Plant sap for example perilla leaf	[72]
6	SC/SPE	SPCE (C110)	PEDOT:PEG	TDANO_3_	−55.8	10^−6^	0.1–1.12 × 10^−6^	90.9 µV/s (I = 10 nA)	4–10	Agricultural growth medium	[73]
7	SC/micro-sized ASS	Cu wire	graphite	TDANO_3_	55.5–58.5	5 × 10^−6^	10^−1^–10^−5^	n.m.	2–7	Microalgal productions	[74]
8	SC/SPE	S3PE	PPy	pTHFA (alternative membrane) and TOANO_3_	−55.3	3.47 × 10^−5^	10^−1^–10^−4^	-	-	Fish ponds water, soil, river water	[75]
9	SC	GCE	Chitosan, black phosphorus, ferric oxide	TDABr	−58.5 ± 0.5	10^−6.2−6^	10^−1^–10^−6^	0.047	-	Agricultural and emvironmental systems	[76]
10	SC/SPE	DuPont 7102 CPPE		TOABr	−57.1–60.1	-	10^−1^–10^−4^	-	-	Agriculture field	[77]
11	SC	Carbon paste, dipropylene glycol dimethyl ether on a PET		TOABr	-	-	-	-	-	Analyzing the degradation of nitrates	[81]
12	SC	AuE	TRGO	Nitrate ionophore VI	−60.0 ± 0.5	4 × 10^−6^	4 × 10^−5^–1 × 10^−1^	-	2–10	blood	[78]
13	SC	CB-hydrous ruthenium dioxide paste electrode		Nitrate ionophore V	−51.1 ± 0.1	10^−5.5±0.06^	10^−1^–10^−5^	0.19 mV/h	2–10	Plant substrates and water samples	[79]
14	SC	GCE	PGCP	PPy-NO_3_	−50.86	4.64 × 10^−6^	1 × 10^−5^–5 × 10^−1^	0.315 µV/s	3.5–9.5	Environmental and clinical samples	[80]
15	LC	ISE body (Sigma 45137-1EA)	1 × 10^−3^ M KCl	TDMACl	−59.5	-	10^−1^–10^−5^	-	-	Nutrient solutions	[68]

**Table 4:** The various types of NO_3_-ISEs without analytical application.

No.	Electrode Type	Working Electrode	Transducer Media	Selectivity Provider	Slope	Limit of Detection	Linearity Range	Stability	pH	Reference
1	SC	GCE	PEDOT-C1_4_	TDMANO_3_	−52.2	10^−5.5^	1 × 10^−1^–5 × 10^−4^	-	-	[82]
2	SC	Ag wire	MCB	TDMANO_3_	−54.8	2.5 × 10^−6^	5 × 10^−5^–1 × 10^−1^	2–10 mV/day	-	[83]
3	SC/SPE	Au electrode	POT-MoS_2_	TDMANO_3_	−55.7	-	10^−1^–10^−4^	-	-	[53]
4	SC/SPE	GrE	PPy	TDMANO_3_	55.6–58.2	-	10^−1^–10^−4^	-	-	[53]
5	SC/SPE	Carbon electrode	PVAc@NaNO_3_ electrolyte layer	TDANO_3_	−58.56	-	10^−1^–10^−5^	2.61 mV/h	-	[84]
6	SC/SPE	CPE		TOANO_3_	−54.0 ± 0.3	Loog_10–_4.48 ± 0.25	1–10^−4^	-	-	[85]
7	SC	GC disc	Graphene	Nitrate ionophore V	54.32 ± 0.39	2.63 × 10^−6^	10^−1^–10^−6^	0.065 mV/h	-	[87]
8	SC	GC disc	CB	Nitrate ionophore V	−54.22 ± 0.09	2.95 × 10^−6^	10^−1^–10^−6^	0.082 mV/h	-	[87]
9	SC	GC disc	CNTs	Nitrate ionophore V	−54.15 ± 0.18	2.31 × 10^−6^	10^−1^–10^−6^	0.087 mV/h	-	[87]
10	SC	Pt electrode	PAAm-MnO_2_	Nitrate ionophore V	−50.6	−10^−5.2^	10^−1^–10^−5.2^	-	-	[86]
11	SC	GCE	Ni-HAB MOF	Nitrate ionophore VI	56.8	6.23 × 10^−6^	10^−1^–10^−4^	1.3 × 10^−4^ µV/h	-	[89]
12	SC	Au-coated plastic interdigitated electrode	PPy	Nitrate ionophore VI	−54.4 ± 1.3	-	0.1–10^−4^	-	-	[88]
13	SC	GCE	Au layer	PPy-NO_3_	54	1.1 × 10^−4^	0.1–10^−4^	-	-	[90]
14	SC	GCE	MWCNTs-THTDPCl	Co(Bphen)_2_(NO_3_)_2_(H_2_O)_2_	−57.1	5 × 10^−7^	1 × 10^−6^–1 × 10^−1^	0.042 µV/s	4.6–10.8	[93]
15	SC/SPE	Mixture of carbon paste and di propylene glycol dimethyl ether PET substrate		-	−48 ± −1.5	10^−5^	10^−1^–10^−4^	0.1 mV/day	-	[92]
16	SC	Carbon electrode (graphite rod or carbon paste containing dry battery waste)			−57.3–60.8	3.2–6.5 × 10^−5^	0.1–10^−4^	-	-	[91]

**Table 5:** Ammonium ISEs used for soil monitoring.

No.	Electrode Type	Working Electrode	Transducer Media	Selectivity Provider	Slope	LOD	Linearity Range	Stability	pH	Reference
1	SC	GCE	MWCNTs:CNFs	nonactin	58.4	2.5 × 10^−6^	10^−1^–10^−5^	0.48 mV/h	3.5–9.3	[95]
2	SC	Graphite line		nonactin	-	5.3 × 10^−6^	5 × 10^−3^–10^−5^	-	-	[44]
3	SC/SPE	Carbon electrode		nonactin	43.04	-	5.5 × 10^−5^–6.82 × 10^−3^	0.3 mV/h	-	[96]
4	SC/SPE	AuE		nonactin	-	-	0–1.8 × 10^−3^	-	-	[97]
5	SC/SPE	AuE		nonactin	53.6 ± 5.1		10^−1^–10^−4^	-	-	[98]

**Table 6:** Comparison of NH_4_-ISEs used for determination of ammonium ions in aqueous samples.

No.	Electrode Type	Working Electrode	Transducer Media	Selectivity Provider	Slope	LOD	Linearity Range	Stability	pH	Reference
1	SC	GCE	AgNPs@MXene	nonactin	51.68	5.89 × 10^−7^	10^−5^–10^−1^	-	-	[99]
2	SC	GCE	AuNPs-rGO	nonactin	56.94 ± 1.57	3.8 × 10^−6^	10^−5^–10^−2^	-	-	[100]
3	SC/SPE	pencil contact pads and Cu contacts	graphene	nonactin	-	4.8 × 10^−6^	3.6 × 10^−5^–3.6 × 10^−3^	-	4–10	[54]
4	SC/SPE	Cu based conductive ink	PEDOT:PSS and MWCNTs	nonactin	59.2	<5.5 × 10^−4^	10^−4^–10^−1^	1.3 mV/h	6–9	[102]
5	SC/ink printed	Injected-printed Ag electrode	GrNPs and polyvinyl butyral	nonactin	57.3	4.8 × 10^−6^	10^−1^–10^−5^	High stability	2.5–8.5	[103]
6	SC/ink printed	Inkjet printing Ag	inkjet-printed melamine-intercalated graphene nanosheets	nonactin	55.59 ± 0.43	0.88 × 10^−6^	10^−1^–10^−6^	-	-	[104]

**Table 7:** ISEs used for monitoring of ammonium ions in various samples.

No	Electrode Type	Working Electrode	Transducer Media	Selectivity Provider	Slope	LOD	Linearity Range	Stability	pH	Sample	Reference
1	SC	GCE	SnO_2_	nonactin	47.17	1.18 × 10^−8^	10^−2^–10^−7^	1.3µV/s (I = 1 nA)	3–8	Aquaponic nutrient solutions	[106]
2	SC	GCE	Chitosan, black phosphorus combined, ferric oxide magnetic nanoparticles	nonactin	57.8 ± 0.1	10^−5.4^	10^−5^–10^−1^	0.064 µV/s	-	Agricultural and environmental systems	[76]
3	SC	CO_2_ LIG		nonactin	51	3 × 10^−5^	1 × 10^−4^–1.5 × 10^−1^	-	3.5–9.0	Urine testing	[107]
4	SC/SPE	AuNPs ink		Nonactin + CNTs	56.2 ± 2.3	-	10^−1^–10^−4^	-	-	Sweat monitoring	[108]
5	SC/SPE	Carbon and Ag/AgCl layers on a PU-PDMS substrate	Gr-CNTs	nonactin	59.6 ± 1.5	<10^−6^	10^−1^–10^−6^	High stability	-	Sweat monitoring	[110]
6	SC/SPE	Carbon ink layer	MWCNTs–COOH	nonactin	55.36	5.01 × 10^−6^	10–60 mM	-	-	Fingertip sweat	[109]
7	SC	PET substrate and AuE layer	PEDOT	nonactin	59.3	-	10^−2^–10^−5^	2–3 mV/h	-	Fresh crude vegetable leaf juices	[105]
8	SC	CB-hydrous ruthenium dioxide paste electrode		nonactin	59.3 ± 0.1	10^−5.09±0.08^	10^−5^–10^−1^	0.23 mV/h	2–8	Plant substrates	[79]

**Table 8:** Comparison of NH_4_-ISEs without analytical application.

No.	Electrode Type	Working Electrode	Transducer Media	Selectivity Provider	Slope	Detection Limit	Linearity Range	Stability	pH	Reference
1	SC	GCE	CuHCF		57.7 ± 0.2	4.2 × 10^−5^	10^−1^–5 × 10^−5^	-	4–10	[111]
2	SC	GCE	CuHCF	Nonactin	56.3 ± 0.1	4.5 × 10^−6^	10^−1^–10^−6^	-	4–10	[111]
3	SC	GF	RACNT directly grown on GF	nonactin	58.2 ± 0.6	7.5 × 10^−6^	10^−1^–10^−5^	-	-	[112]

## Data Availability

Data will be available upon request.

## Abbreviations

AgNPs@MXene	Ag nanoparticles on the surface of two-dimensional transition metal car bides
AI	artificial inteligence
ASS	all-solid-state
ATP	adenosine-5′-triphosphate
AuE	Gold electrode
AuNPs	gold nanoparticles
CAGE-1	1,3,5-tri(p-hydroxyphenyl)benzene-based chlorotriazine pillared cage molecule
CB	carbon black
CNFs	carbon nanofibers
CNTs	carbon nanotubes
MWCNTs-COOH	Carboxylated multi–walled carbon nanotubes
CoWSe2	bimmetalic sellenium compounds
CPE	carbon paste electrode
CPPE	carbon paste printed electrode
CRGN	chemically reduced graphene
CuHCF	copper(II)-hexacyanoferrate
CuONPs	copper oxide nanoparticles
DNA	deoxyribonucleic acid
ERGO	electrochemically reduced graphene oxide
FETs	field effect transistors
FIA	flow injection analysis
f-SWCNTs	octadecyl amine-functionalized single-walled carbon nanotubes
GC	glassy carbon
GCE	glassy carbon electrode
GD	glass fiber
GO	graphite oxide
GrE	graphite electrode
IE	internal electrolyte
ISE	ion-selective electrode
ISFETs	ion sensitive field effect transistors
ISM	ion-selective membrane
IoT	internet of things
LC	liquid contact
LCISE	liquid contact ion-selective electrode
LIG	laser induced graphene
LOD	limit of detection
LR	linearity range
MCB	mesoporous carbon
MWCNTs	multi-walled carbon nanotubes
Ni-HAB MOF	high-capacity metal-organic framework
NPOE	2-Nitrophenyl octyl ether
PAAm-MnO2	manganese dioxide and poly(allylamine) composite
PANI	polyaniline
PANINFs-Cl	polyaniline doped with chloride ions
PANINFs-NO3	polyaniline doped with nitrate ions
PDMS	poly(dimethylsiloxane)
PEDOT	poly(3,4-ethylenedioxythiophene)
PEG	polyethylene glycol
PET	polyethylene terephthalate
PGCP	pericarpium granati-derived biochar with phosphoric acid activation
PPy	polypyrrole
POT	poly(3-octylthiphene-2,5-diyl)
PSS	polystyrene sulfonate
PTFE	poly(tetrafluoroethylene)
pTHFA	photocurable poly-tetrahydrofurfuryl acrylate
PU	polyurethane
PVAc	poly(vinyl acetate)
PVC	poly(vinyl) chloride
RACNT	radially aligned carbon nanotube
RE	reference electrode
rGOA	reduced graphene oxide aerogel
RNA	ribonucleic acid
RSD	relative standard deviation
S3PE	single strip screen-printed electrode
SC	solid contact
SCISE	solid contact ion-selective electrode
SPCE	screen-printed carbon electrode
SPE	screen-printed electrode
TDA+	quaternary ammonium cation
TDABr	tetradodecylammonium bromide
TDACl	tetradodecylammonium chloride
TDANO3	tetradodecylammonium nitrate
TDMANO3	tridodecylmethylammonium nitrate
THANO3	tetraheptylammonium nitrate
THTDPCl	ionic liquid trihexyl(tetradecyl)phosphonium chloride
TOABr	tetraoctylammonium bromide
TOANO3	tetraoctylammonium nitrate
TPM	3-(trimethoxysilyl)propyl methacrylate
TRGO	thiol-functionalized reduced graphene oxide
WHO	World Health Organisation
